# St. Thomas's Hospital

**Published:** 1831-01-01

**Authors:** 


### ST. THOMAS'S HOSPITAL.

### PARALYSIS AGITANS.

IN the fourth volume of the Medico-Chirurgical Journal, for November, 1817, we gave a full account of a pamphlet published by the late Mr. Parkinson, of Hoxton, on a peculiar disease, which he denominated "PARALYSIS AGITANS," and which, though little noticed, cannot be very rare, since, at this moment, we have three patients under our care, afflicted with the complaint—two males and one female.

So imperceptible is the approach of this malady, that the precise period of its commencement is seldom recollected by the patient. A slight sense of weakness, with a proneness to trembling, sometimes in the head, but most commonly in the hands or arms, are the first symptoms noticed. These affections gradually increase, and, at the period perhaps of twelve months from their first being observed, the patient, particularly while walking, bends himself forward. Soon after this, his legs suffer similar agitations and loss of power with the hands and arms.

As the disease advances, the limbs become less and less capable of executing the dictates of the will, while the unhappy sufferer seldom experiences even a few minutes suspension of the tremulous agitation; and should it be stopped in one limb, by a sudden change of posture, it soon makes its appearance in another. Walking, as it diverts his attention from unpleasant reflections, is a mode of exercise to which the patient is in general very partial. Of this temporary mitigation of suffering, however, he is soon deprived. When he attempts to advance, he is thrown on the toes and fore part of his feet, and impelled, unwillingly, to adopt a running pace; is in danger of falling on his face at every step. In the more advanced stage of the disease, the tremulous motions of the limbs occur during sleep, and augment in violence until they awaken the patient in much agitation and alarm. The power of conveying the food to the mouth is impeded, so that he must submit to be fed by others. The torpid bowels require stimulating medicine to excite them into action. Mechanical aid is often necessary to remove the fæces from the rectum. The trunk is permanently bowed. Muscular power is diminished. Mastication and deglutition are difficult; and the saliva constantly dribbles from the mouth. The agitation now becomes more vehement and constant; and when exhausted nature seizes a small portion of sleep, its violence is such as to shake the whole room. The chin is almost immoveably bent down upon the sternum; the power of articulation is lost; the urine and fæces are discharged involuntarily; and coma, with slight delirium, closes the scene.

In a clinical lecture lately delivered by Dr. Elliotson, at St. Thomas's Hospital a case of this description is introduced as a hook on which to hang some practical and pathological remarks—the case is as follows.

"The patient, F. E., is 38 years of age, and has had the disease eighteen months. He has been accustomed to drink hard at different periods of his life. He is a school-master by profession. It is the right upper extremity which is now affected; but though the right lower extremity is not in agitation, it is occasionally retracted as he walks,—experiences solitary catchings, though it does not shake. The disease began in the head and tongue, but when the right upper extremity was affected, it left the head. This peculiarity distinguishes the present case, that the tongue is one of the parts that were first affected. In general this is not the case, and the tongue is not affected, after many other parts have suffered severely. The head now shakes very slightly only. The affection of the tongue is attended by the following very curious result. Whenever the man attempts to speak, the tongue begins to quiver like the tongue of a serpent; presently a confused murmur is heard, and then suddenly he brings out his words with extreme rapidity; and such is the effort that he cannot stop himself but repeats the few last words again and again. It is a phenomenon analogous to the running which occurs on the attempt to walk. He cannot manage the muscles at all, without a violent effort, such an effort that his tongue gets as it were into a run; the common expression of the tongue running, when we describe a person who makes a good use of it, is really applicable to this patient. I have written down in the case-book, 'Before he can speak he makes a confused and inarticulate murmur, and then speaks rapidly, slurring his words together, and repeating the last words several times. The effort makes the tongue and right upper extremity shake violently.' He sleeps very well, his appetite is good, and in all other respects, except this shaking of the body, he is in tolerable health. Sometimes after a good night's rest he does not shake at all for a few minutes after waking in the morning, but then it is not long before the trembling commences. Any excitement or attempt to do any thing at once, greatly increases the tremors, but by a strong effort he can at length arrest them for a few moments. The only other symptom present is costiveness (he has but two stools a week), and a pain in the head whenever he is anxious."*

Dr. Elliotson informs us that, in many cases, the disease is controllable by art—"it ceases on active treatment." In young persons he has often cured it—and, in such cases, there has been great constipation. It has so happened that all the people we have seen affected with this complaint, have been advanced in life, and therefore our practice has not been so successful as that of Dr. Elliotson. Yet we have sometimes seen considerable relief produced by medicine. One of the patients now under our care, a music-seller on Holborn-hill, came under our observation about five months ago, and was then so ill that we entertained no hope of doing any good. Regular aperients were prescribed, together with colchicum and bitters. We heard no more of him

---

* See the Lancet and the Medical Gazette for Oct. 16, 1830.

till within these few weeks, when, being summoned to him, we found him in nearly the same state as on our first visit. But we were rather surprised to learn that, soon after he commenced the remedies above-mentioned, he got so much better that he almost daily went out—sometimes as far as Hampstead. This man has an insatiable appetite, and the indulgence of this appetite has, no doubt, an injurious effect on the complaint. We shall introduce the following remarks of Dr. Elliotson on the treatment.

"If we can ascertain that there is any fulness in any part of the nervous system, or any inflammation, the treatment should consist in bleeding locally or generally, or both—in purging and mercurializing—in employing setons, issues, moxas, &c. If there be nothing of this kind—if there be no reason to suspect fulness, or inflammation—if the patient be not of a plethoric habit, and no local pain nor tenderness be felt, then such treatment is, for the most part, inefficacious. I would, therefore, not have recourse to treatment of this description unless there was a plethoric habit, or evident marks of inflammation, or fulness in some part of the nervous system; or unless there had been some injury, the effect of which we should necessarily suppose to be chronic inflammation. Almost all nervous diseases, whether convulsive, spasmodic, or paralytic, may arise from, or be dependent upon, inflammation or congestion, or upon some peculiar state which we do not understand. I know of no mode of distinguishing these varieties of the disease, except what I have already pointed out. When we cannot ascertain that the disease has arisen from mechanical injury, and there is no local pain or tenderness, or fulness of the system, stimulants, tonics, electricity, the shower bath, and various remedies—the operation of which we do not understand — iron, sulphate of zinc, copper, nitrate of silver, and in short all those minerals which belong to a class of remedies, each of which do good, and has a peculiar operation on the nervous system, distinct from that of narcotics, perfectly inexplicable—often prove efficacious in this and all other convulsive, spasmodic, and paralytic diseases. The present patient appears to have been both in the St. George's and Middlesex Hospitals, and from what I have learned of his previous treatment I have directed the plan which he is now undergoing. I find that, very rationally and properly, in St. George's Hospital, he had been cupped and bled frequently; that counter-irritation had been produced by means of blisters, so that a copious discharge was kept up from the back of the head and neck; and that he had been kept on low diet. The plan which it was reasonable to pursue in such a case, where the patient was in the prime of life, the habit full, and a blow had occurred—this general and local antiphlogistic treatment, which is often successful in nervous diseases, was fully pursued, but in vain. I find likewise that in the Middlesex Hospital, it having been ascertained that these means had proved unsuccessful after full trial, stimulants and tonics were administered to him — porter, good nourishment, camphor, and various stimulant remedies, and although these did not cure him, this mode of treatment was as fully justified after the former, as the former was in the first instance. As he was of full habit the treatment began with antiphlogistic means, and in failure of them recourse was had to stimulants. Among the various remedies which do good in the diversified diseases of the nervous system, I believe the most valuable, and at the same time the most safe, is *iron*. Upon the whole I have succeeded better with that than with any others, though in epilepsy it rarely does good. I have been much more successful in the treatment of St. Vitus's dance with iron than with any other internal mineral remedies, although their efficacy cannot be doubted. It is far less nauseating and griping than copper; it does not produce the same inconveniences that arise from arsenic, nor the sickness which results from sulphate of zinc, nor does it produce that blackness of skin which is the effect of nitrate of silver, and the chance of which makes me always unwilling to employ it. Knowing, therefore, the treatment which the patient had previously undergone, and hearing from him that he was always better the more he was strengthened, I ordered him two drachms of subcarbonate of iron three times a day, and a pint of porter at dinner. He is a superior sort of man, and very desirous of recovery, and I do not imagine that he would deceive me by telling me he was better for invigorating measures, if he were not. It is certainly necessary to remedy the state of costiveness under which he labours, but I do not suppose that purging would cure his disease, though I am perfectly aware that, where there is congestion or inflammation, or an approach to it, purging frequently does cure patients with various nervous disorders. But on the whole I really have been disappointed in the use of purgatives, though I acknowledge their value in various diseases of the nervous system. If there be no reason in this case to suppose congestion or inflammation, still the bowels are costive, and that is a state to be remedied, for constipation must make the disease worse. Under these circumstances I have ordered him to take half a minim of croton oil daily, in order to keep his bowels freely open; for though purging him may do him no good, and by debilitating would probably make him worse, yet constipation will be sure to do him harm. There is another reason also why costiveness should be obviated;—the iron would be liable to accumulate in the alimentary canal. The carbonate is a bulky remedy, and if any deficiency of the alvine discharge occurs, it will of course accumulate. I usually administer it in treacle, because treacle has a tendency to keep the bowels open."*

As Mr. Parkinson's pamphlet is now almost forgotten, and perhaps was never in the hands of many of our readers, we shall here introduce a brief abstract of a few of his cases.

*Case* 1. A gardener, aged 50, remarkably sober and temperate, who had never suffered from rheumatism, pains in the head, or any apoplectic seizure, first noticed a slight trembling of his left hand and arm, after more than ordinary exertion. In the progress of this case, every circumstance mentioned in the preceding history occurred.

*Case* 2. A man, 64 years of age, casually met in the street, had suffered from the disease about eight or ten years. He described it as having come on very gradually, and attributed it to irregularities in living, but particularly to indulgence in spirituous liquors. All the extremities were agitated; the speech interrupted; and the body bowed and shaken. He walked so entirely on the fore part of his feet, that he would have fallen, had it not been for the assistance of a stick.

*Case* 3. The subject of this case was also met in the street. He was a man of a remarkably athletic frame, 65 years of age. The agitation of the limbs, head, and whole body, was too violent to allow it to be designated as *trembling*. The body was bowed, and the head thrown so forward as to oblige the man to go on a continual run, and employ his stick every five or six steps, to force himself into an upright posture. The man stated he had been a sailor, and thought his complaint arose from lying on the bare earth during a confinement of some months in a Spanish prison.

*Case* 4. A gentleman, ætat. 55, who had experienced a trembling of his arms for about five years, had inflammation of the side, which terminated in an abscess, and discharged a quantity of matter daily for two or three weeks, without effecting any alteration in his original complaint.

*Case* 5. A gentleman was seen at a distance supported by an attendant, who was standing before him with a hand on each shoulder. By gently swaying backwards and forwards, he placed himself in equipoise; and, giving the word, started in a running pace, the attendant gliding before him, ready to receive him and prevent his falling after having run about twenty paces.

*Case* 6. A gentleman, ætat. 72, has

* Med. Gazette, No. 150, p. 91-2.

suffered from the disease some years. Twelve months since, he had hemiplegia of the right side, which was entirely removed in a fortnight, by purgatives, and a blister behind the neck. At present, he is almost constantly troubled with the agitation, but is generally able to stop it for a short period by a sudden change of posture, such, for instance, as throwing himself into a chair. His sleep is much disturbed; he is scarcely able to feed himself; and when he walks, it is with the constant apprehension of falling forwards.

The author is of opinion, that the preceding are all cases of the same disease, differing only in stage and duration.

Reverting to Dr. Elliotson, that talented physician cautions us against confounding paralysis agitans with the tremulous agitations of drunkards. The latter generally affects both hands, and commences or increases on any muscular effort. In paralysis agitans, the circumstances are just the reverse—a strong effort will, for a few moments, suspend the tremor.

---

### XVII.

### WESTMINSTER HOSPITAL.

### FATAL CYNANCHE.

### XLIV.

### ST. THOMAS'S HOSPITAL.

*Surgical.*

### CASES OF INJURY TO THE SPINE.

CASE 1. *Fracture of Bodies and Spinous Processes of Dorsal Vertebræ, without Depression—Dislocation of the Heads of Six Ribs—Fracture of Scapula —Effusion of Blood into the Thorax from Laceration of the Vertebral Vein.*

George Bilson, æt. 27, a brewer's drayman, very tall and muscular, was brought into the hospital about seven o'clock, p.m. on the 29th of October, a sack of hops of immense weight (said to be 4 cwt.) having fallen upon him from a considerable height about half an hour previously, in consequence of the crane having given way. He had lost all power of motion as well as sensation, and appeared labouring under symptoms of severe injury to the brain, still however retaining consciousness of pain, as, when placed in bed, he frequently desired to be "lifted up." Pupils were dilated and insensible to light. He had been bled before admission, and the pulse was slow, 60, small, and weak —breathing laborious, short, and frequent, not stertorous—lower extremities cold. Brandy and water were administered by the dresser, and hot flannels applied to the legs and feet. Faintness, occasional vomiting, and other symptoms of collapse were present until he was seen by Mr. Tyrrell, about ten o'clock. It was evident to Mr. T. that nothing but a fatal termination could be anticipated, and that very speedily. He directed as much brandy to be given in water at frequent intervals as the patient could be got to swallow, and the whole body to be kept warm. Death took place at ¼ past one, rather less than seven hours from the injury, the system not having rallied from the shock it had sustained.

*Examination of the body 36 hours after death.* In the right pleural sac was found a pint of uncoagulated venous blood, which had escaped through a rent in the right vertebral vein, produced by a spiculum of the first rib, which was obliquely fractured. The heads of six ribs, from the third to the ninth inclusive, were torn from their articulations with the bodies of the corresponding vertebræ, and projected considerably forwards, fully an inch, into the posterior mediastinum, in the cellular membrane of which there was also effused a quantity of venous blood from the same source. A fracture had occurred in a transverse direction through the body of the fourth dorsal vertebra, by which the spine was completely divided. A portion of the anterior part of the fifth was also torn off along with the lower piece of the fourth. The four spinous processes in the immediate vicinity, with the arch of the fourth, were detached from their vertebræ. The ligamentum subflavum and intervertebral substance of these two bones were both torn asunder, and the articular surfaces of both were also literally smashed, no traces of them being left in their natural situation. The texture of the spinal marrow was here entirely disorganized, but at the time of examination it was not considered that any compression had existed. Hence we may infer that the severe injury of the bony structure produced the disorganization of the chord at the time of the accident. Some effusion of dark blood was found to occupy a considerable surface of the cerebrum and cerebellum, beneath the arachnoid membrane. Much injury was done to the scapula. The acromion was broken about half an inch from its attachment to the clavicle, and the spine of the bone was separated from the venter, excepting its connexion by periosteum. Fractures, or rather fissures, passed through both supra and infra-spinal fossæ.

CASE 2. *Fracture and Dislocation of Cervical Vertebra—Laceration of Intervertebral Substance, and of Vertebral Artery.*

William Thompson, ætat. 30, excessively corpulent, was admitted into King's Ward, April 12th, 1830, three hours after he met with the following accident. The person who brought him stated that he had fallen over the side of a cart he was driving, (being drunk at the time,) and that, in falling, he struck his head against the wheel. A large angular portion of the scalp was torn up on the right side. Although he was intoxicated on admission, it was evident that there was some graver cause for his symptoms than mere inebriety; he had not the slightest power over his extremities, and complained of excessive pain at the back of his neck, when touched; the total inability to keep his head supported by his own efforts was also very remarkable. His face was much flushed—pulse slow and full—breathing carried on by the diaphragm *alone.*

April 13th. During the night he passed his fæces involuntarily—his urine was retained—penis in a state of partial erection—limbs, both upper and lower, insensible and powerless—he was quite unable to raise his head, and complained of great pain in the neck, where there was most acute tenderness on pressure. Ordered hirud. xx. to the nape of the neck, and a colocynth enema to be thrown up immediately.

His breathing was quick during this day, but on the morning of the 14th it became suddenly oppressed, and he died in the afternoon of the same day gasping for breath, as though his power of inspiring air was completely exhausted.

On examination it was found that the fourth cervical vertebra was extensively fractured, all the processes being broken from the body and divided into numerous pieces. The intervertebral substance between it and the fifth was torn through, and one of the articular surfaces driven from its correspondent one, by which there was a partial dislocation forwards of the body of the fourth. The medulla spinalis was much softened in the situation of the injury, and there was a large quantity of blood extravasated amongst the muscles and into the vertebral canal from the laceration of the right vertebral artery. The wound of the scalp had nearly united by lymph.

The superior origin (viz. from the third cervical) of the phrenic nerve being here left entire, it would appear that the diaphragm acted through it alone, but this muscle, being deprived of the greater supply of nervous influence from the fourth nerve, was at length unable to continue its office.

CASE 3. *Fracture of Thigh just above the Condyles—Wound of the Soft Parts communicating with the Knee-joint—Lacerated Wound of opposite Ancle—Death—Tubercles in the Lungs.*

John Southcoat, æt. 46, admitted in the middle of the day on Oct. 4th, 1830. This man had been addicted to drinking and looked very old for his years.

The right leg and foot were everted, and the femur was found to be fractured obliquely a little above the condyles, in direction downwards, and slightly inwards. A deep punctured wound, nearly an inch in length, was also observed on the outer and back part of the thigh, about two inches above the outer condyle. There was likewise a lacerated wound of the left foot, a flap of integument being turned up of an irregular shape and about six inches in length, passing from the outer ancle downwards and forwards on the dorsum pedis. This was brought together by sutures and straps, and strapping was applied to the preceding. Both limbs were properly supported on pillows in a position easy to the patient, who was considerably exhausted, and cold lotion was applied in the neighbourhood of the fracture.

On the following day the limb was put up in Mr. Amesbury's fracture apparatus for the lower extremity, the leg being flexed on the thigh, which was elevated, and the patient placed on his back. An evaporating lotion was kept on the knee, which was rather swollen, but neither red nor painful. Pulse 96, soft. Bowels open by medicine. Oct. 7th. Uneasiness during the night from what he considered distention of the bladder—urine has been dribbling away from the penis—the catheter being introduced afforded some relief, although very little urine was drawn off. Pulse 100, and soft—tongue moist—skin rather dry and warmer than natural—bowels open.

8th. Limb easy—no starting has taken place—wound on the foot has slight superficial sloughs, and its edges are separated—two sutures were cut away, and fresh straps lightly put on, with poultice. Patient had little sleep in the night, and is troubled with a hacking cough—no expectoration—free from pain of side or chest, excepting that produced by coughing—has had, however, attacks of pain here—has taken no nourishment—pulse smaller, 108—tongue rather dry and yellowish—bowels open, and urine is voided freely. Ordered beef-tea.

9th. Pulse 108, not so feeble—tongue yellower—skin rather dry—urine easily voided—bowels opened more than once yesterday—swelling has taken place about the knee, but it is now less, and both limbs are quite easy.

11th. Diarrhœa since yesterday—motions very frequent, but not passed with much pain—sickness, but no vomiting—belly soft, not tender—tongue brown, furred, and dry, particularly in the centre—pulse very small and weak—countenance sunken. Had leeches to the knee yesterday, as this had become more swollen; it has now rather a livid appearance from ecchymosis, and is considerably puffed—wound of left foot sloughy, surrounded for some distance by redness. The dresser ordered tinct. opii, ℳviij. inf. catech. co. ℥j. 2dis horis, and the patient shortly rallied in a surprising degree. On removing the poultice from the wound in the thigh, above an ounce of clear synovial fluid escaped.

In the evening diarrhœa returned with vomiting and constant retching—the powers of life were much more fallen. He was seen by Mr. Green, who ordered two grains of opium to be given immediately—brandy and arrowroot—and a blister to the scrobiculus cordis.

12th. Vomiting was somewhat allayed by the opium, but after a few hours it returned with the same painful retching. The remedy was accordingly repeated in the night about one o'clock. Has not been so much disturbed in his bowels during the morning, and there is no tenderness or swelling of the abdomen—but this is quite supple—has taken egg and brandy in some quantity, which has fortunately staid on his stomach. Pulse very low at present—tongue more brown, its entire surface being now involved—feet and hands cool—cough very distressing—no sputa—countenance not so haggard, nor features so contracted—a flapping motion of the lips and cheeks, as if he was blowing something away from them; a symptom we have observed uniformly unfavourable. Blister is placed rather high up, and has not yet risen. ℞. Tinct. opii, ♏lxxx. Decoct. amyli, ℥ij. ft. enema statim. injiciend. Brandy to be taken in cold arrow-root.

The poor patient gradually sunk from this time, and expired at half-past six on the following morning.

*Examination of the body 30 hours after death.* The external wound of the thigh was found to communicate with the cavity of the knee-joint, by means of an opening in the capsule in front, about ¼ of an inch in diameter, just above the articular surface of the internal condyle. This laceration was evidently produced by a detached spiculum from the upper fractured extremity, immediately contiguous. The femur was fractured obliquely as to the thickness of the bone, not as to its shaft. The surfaces of the fracture were from two to three inches in vertical length, commencing posteriorly and opposite the integumental wound and terminating immediately above the articular surfaces of the femur, the lower edge of the upper fractured extremity being here acute, and the femur thus presenting the appearance as if a lamina of bone had been mechanically scaled off. A good deal of coagulum was effused around and between the fractured ends, the result of injury.

*Chest.* Bronchia filled with frothy mucus at their root, and reddened in their internal surface. Upper parts of superior lobes of both lungs contained large and solid tubercles, which appearances easily explain the origin of the cough so much complained of during life. One or two atheromatous patches were observed in the arch of the aorta, the inner surface of which was also of a dark red colour for the greater part of its extent. This was considered merely a stain, as the mucous coat of the vessel was in some parts quite natural in structure.

*Abdomen.* Liver hardened in texture, rather inclined to a nutmeg brown, but not enlarged. Internal surface of stomach rather florid at the left extremity, and also somewhat hardened at the pylorus, the usual annular feel of which was lost. Here we probably see the cause of the vomiting and purging, as scarcely any disease existed in the rest of the alimentary canal. Both muscular and mucous coats of bladder thickened; the former more especially, which was of a hard texture—there was some redness of the latter membrane. It was rather singular that the patient at one time had the uneasiness in hypogastric region and dribbling of urine from the penis, mentioned in the report, which were effectually relieved by the introduction of the catheter, although the bladder contained very little urine.

CASE 4. *Simple Fracture of the Leg, becoming Compound-Non-union in consequence of Necrosis.*

John M'Canne, æt. 38, a strong robust sailor, was admitted under Mr. Green, January 5th, 1830, with an oblique fracture of the tibia downwards and inwards near its middle, and fracture of the fibula a little lower down. The injury was caused by the falling of a piece of timber on the front of the leg. Crepitus was exceedingly distinct, and there was considerable bruising of the integuments. The leg was laid upon a pillow, with the heel carefully raised to counteract the pressure of the upper fractured end of bone against the skin. Hirud. xvj. lot. spirituos.

6th. To-day the leg was put up in Mr. Amesbury's apparatus for simple fracture. The apposition seemed perfect, and the limb was of the same length as its fellow. Some redness and heat were observed in the injured parts, to which the lotion was continued. Some slight feverish symptoms were present, as loss of appetite, pulse full, and rather sharp, dry tongue, but not in any excess.

7th. The leech-bites have ulcerated, and discharge a thin purulent fluid. The spirit wash to be left off, and cerat. simplex applied. He continued much in the same state till the—

13th, when the feverish symptoms had greatly increased—countenance was flushed, tongue more furred, pulse quick and sharp, urine scanty and high-coloured—no stool since the 11th. An ill-conditioned sore had formed in the neighbourhood of the fracture, with sloughy edges surrounded by considerable redness, and affording a copious discharge, Ordered, Scam. c. Hydr. gr. xvj. statim. Mist. pot. citrat. 4th quâ. horâ. Fever diet—bread poultice to be applied to the wound, with cerat. calaminæ to the bottom part.

14th. Bowels have been freely opened—great sickness at stomach, and pain in the head—thirst—pulse full and sharp, 120. On examination with a probe, a communication is discovered leading to the bone, which is denuded for some extent.

15th. The compound fracture apparatus (which differs from the former in allowing the limb to be placed in the straight or bent position, as may be found necessary) was to-day substituted for the simple, and the leg extended—wound appears more sloughy. ℞. Mist. camph.℥iss., Ammon.carb. gr. v., Tinct. opii, ♏v. 6tis—sago and syrup. Port wine, ℥ij. in the day. Cat. lini.

17th. Leg easy—pain in chest and dyspnœa—had yesterday a slight attack of erysipelas of the face, which has in some degree subsided. Tongue dry—pulse quick, not full, or hard. Empl. lyttæ pectori. Demulcent mixture to be taken occasionally.

18th. Relieved from pain and dyspnœa—otherwise the same.

20th. Going on well—swelling of the face less—sloughing extending in the leg, but not deeply—medicine to be taken every four hours. Lot. chl. calcis, with the poultices.

23d. Has rather troublesome cough—tongue less furred, and moist—pulse 80, small—bowels open—face free from swelling and redness—sloughs of cellular membrane are coming away from each malleolus. Proportions of ammon. carb. and tinct. opii doubled. To have a mutton-chop and a pint of porter daily—continue the wine.

29th. Much the same, but evidently gets weaker, and no sleep at night. To omit his present medicines. Sulph. quin. gr. iij., Inf. ros. co. ℥j., 6tis horis. Opii, gr.j. o. n.

Feb. 2d. No change worthy of mention. Opium, two grains every night.

March 1st. Has been going on without any amendment since last report; does not improve in strength—has no fever—appetite good. No union of the bone has taken place, and a probe introduced through the wound comes in contact with naked bone. The wound presents a healthy granulating appearance, and has much contracted its dimensions. A sore has broken out on the heel from the pressure of the apparatus, which is ordered to be removed, and the leg laid on a pillow. Sores to be dressed with cer. calaminæ.

May 14th. Patient is now quite well in health—sores discharge much less, and are very small. It is, however, quite evident from examination with the probe that the whole cylinder of the bone is dead, and consequently no union can be expected. Amputation has been proposed, and readily assented to by the patient.

21st. The leg was removed by the common circular operation. The skin and cellular membrane were very remarkably thickened—8 ligatures were applied.

22d. Slept for a couple of hours in the night, and has been easy since the operation. Complains, at present, of pain over the tubercle of the tibia. Pulse a little quickened, rather weak—bowels open this morning.

27th. Has gone on well—is put on house-diet, without beer. Part of the dressings removed, and the union of the stump seems perfect.

June 20th. All the ligatures have been cast off a week. As he was this morning endeavouring to walk with crutches, he became suddenly giddy and fell. The stump was torn in front, opposite the spine of tibia, and bled for nearly two hours—the hæmorrhage was ultimately stopped by cold and pressure. It did not return, and the wound healed in a short time.

July 25th. Patient presented well.

*Dissection of the Leg.* Great thickening of the integuments. Muscles wasted. Effusion of fluid into the cellular tissue of the limb. No union had occurred between the broken ends of the tibia in consequence of a large portion of the inferior fractured extremity having become necrosed. The dead part was between two and three inches in length, being nearly detached from the sound bone below, in a direction parallel with the fracture. A shell of bone, a line in thickness, was thus formed from the entire shaft, interposed between the upper extremity and the sound part of that to which it adhered. There was a small abscess between the fractured extremities of the fibula, which contained several minute portions of dead bone. The restorative efforts of nature were, nevertheless, beautifully displayed in the deposition of callus between the fractured ends of tibia and fibula above, and also from the tibia in front to the lower portion; so slight here, however, that it gave way during the examination.

CASE 5. *Malformation of the Heart —Communication between Pulmonary Artery and Aorta.*

Eliza Plumber, æt. 20, admitted as patient of Mr. Green, July 22d, having œdema of the lower extremities with symptoms of disease, or malformation of the heart. She experienced great difficulty of breathing after any exertion, accompanied with palpitation, often long-continued. When she was quiet, the breathing was rather more frequent than natural, but not at all oppressed. She was disinclined to exercise, but could walk about and was able to engage in little domestic concerns. The face and hands were of a purple hue, particularly at the ends of the fingers, in the lips, nose, and cheeks, indicating a mixture of venous with arterial blood. There was constantly coldness of the extremities. She stated that she had laboured under these symptoms as long as she could remember, but could not precisely say whether they had existed from birth, there being a degree of simplicity or want of intellect about the poor girl. The pulse was very feeble, often scarcely to be felt, irregular and intermittent, about 110. The stethoscope applied to the heart did not throw any light on the nature of the affection. Menstruation had never taken place. Ordered, Pil. aloës c. Myrrhâ, gr. v. o. n. Acid. muriat. ♏iv. Syrup. am. ʒj. Decoct. cinch. ℥iss. t. d.

She felt better for some time under this treatment, and the swollen feet and ancles were reduced in size. At intervals she suffered from palpitation and dyspnœa, and the feet then swelled more. In September she took the mist. ferri co. but towards the latter end of the month the blueness and coldness of the hands and feet increased. Her face ultimately had quite a livid appearance. On the 16th October she was attacked with acute pain in her side, and died suddenly in the afternoon.

*Post-mortem appearances.* The heart was much enlarged generally. The walls of the left ventricle in particular, which were in a state of active hypertrophy. A direct communication, capable of admitting a quill, existed between the pulmonary artery and the arch of the aorta, just beyond the origin of the left subclavian, no canal intervening. The ductus arteriosus in the natural situation was deficient, but the very singular malformation we have here placed on record supplied its place in the fœtal state, and this continuing open during life, readily explained all the symptoms then manifested.

There was no disease in the chest or abdomen. BETA.

---

MEDICAL.

CASE 1. *Inflammation of the Brain, excited by a Portion of depressed Bones, a length of time after the Accident.*

Thomas Russel, aged 17, a sailor, was admitted into St. Thomas's Hospital Dec. 5th, 1829, for symptoms so much resembling those of ordinary fever, that his case was believed to be, and treated as such. He had considerable pain in his head, especially on the right side, his face was flushed, pulse quick, and skin hot; he had moreover much pain in his abdomen: there was also a strangeness of manner about him which was unaccountable. These symptoms had come on two days previous to his presenting himself, but no cause could be assigned for their accession by the person who accompanied him; however, from the usual habits of this class of individuals, and the fact of his having received his pay, from the ship to which he belonged, only a few days before, there is a strong presumption that he had been drinking. At this time, indeed, until his death, nothing was said about a *blow* which he had received on his head—judging the case therefore to be one of fever, he was ordered hirud. xij. temporibus. Pulv. rhei c. Hydr. gr. xv. statim sumend. Hydr. c. cretâ, gr. v. ter in die. The head to be shaved and cold lotion applied—and milk diet.

6th. He was somewhat better this morning, but still complained of great pain in his head, which he constantly supported with his hand leaning over the edge of the bed—the pain in the abdomen also continued as before. He was therefore ordered hirud. xx. temp. et hirud. x. regioni cæci. Lotio ammon. acetatis capiti and hydr. c. creta, gr. v. 6tis horis. These means were immediately put in force, but during the day he became worse; two or three severe attacks of convulsions occurred, and towards the afternoon he had some indistinctness of vision. In this state he continued until six o'clock in the evening, when he expired without the slightest struggle; and so suddenly, that before the nurse could bring him some tea he had expressed a wish for, he was dead.

*Post-mortem Examination* 18 *hours after Death.* A cicatrix was now, for the first time, observed in the integuments of the forehead, immediately above the external angular process of the right side. Under this scar an oval portion of the os frontis, equal in size to the disk of a half-crown piece, was found uneven, and slightly depressed: the fractured portion (excepting one point) had re-united. The calvarium was now removed, and it was found, as frequently happens, that the inner table of the bone was more extensively broken than the outer. Three-fourths of this fracture was also re-united, but the remainder, which corresponded to the *point* above specified, projected towards the brain, and presented a very sharp and irregular edge—to fill up the interval thus left between the broken edges, a small portion of new bone had been deposited internally, but in consequence of its irregular growth and prominence it had become a source of irritation. The dura mater was most closely adherent to it, was a good deal puckered, and also thinned; but the most interesting appearance, which at once explained all the peculiarities of the case, was the state of the brain in the immediate vicinity. The membranes, for a space of an inch in diameter, covering the anterior cerebral lobe of the right side, were highly injected, and somewhat thickened—a sensation was communicated to the fingers, as if a tumour had been formed in this part of the brain; its size was equal to a large walnut, well defined, and remarkably firm. A horizontal section having been made, these appearances were found to depend upon coagulable lymph effused in the medullary substance of the hemisphere; the extent of effusion being well defined by a line of increased redness. The colour of the deposit was a deep yellow—its section smooth, and very much firmer than the rest of the cerebral structure—its extent was not greater than was communicated to the fingers before cutting—the cineritious structure in front of the deposit had become very tough and dense—neither on the surface or in any part of the deposit had pus as yet been secreted; but it is highly probable that these appearances were the commencement of an ordinary abscess, which would most likely have become matured but for the sudden termination of life.

The abdominal and thoracic viscera were all healthy.

CASE 2. *Disease of the Internal Ear*

—*Abscesses connected with the Temporal Bone—Abscess of the Brain bursting into the Lateral Ventricle.*

Edgar Barton, ætat. 24, of spare habit, was admitted into St. Thomas's Hospital, December 11th, 1829, on account of discharge of pus from his left ear, with pain of the same side of the head, and deafness. He did not seem at the time to be suffering much, but his face was rather anxious, and he was apparently emaciated. On the 13th, when more particularly examined, he had some feverish symptoms—there was slight diffused swelling immediately above the left ear, and pressure in this situation gave him exquisite pain. The history he gave was, that he had *only been ill a fortnight*—that the discharge from his ear had not existed longer than that time, but that he had suffered (within the same period) most severe pain of the head, more especially above the left ear. A puncture was made into the swelling, through which a small quantity of offensive pus was evacuated; this gave him relief. He was ordered to take mist. citratis potassæ 6tis horis. Pulv. ipecac. comp. gr. x. nocte, and to apply a poultice to the puncture. Towards the evening he became very restless, and during the night he was delirious.

14th. He was now highly delirious, at times so violent that it was necessary to confine him; and when not so, was constantly muttering. He seemed only conscious of extreme pain, for he often pointed to his head, and attempted to pull off the poultice. His skin hot and dry, pulse 100, hard and full—tongue brown and furred. The sense of fluctuation in the part already punctured was now very marked—an incision was therefore made down to the bone, and exit given to about an ounce of extremely fetid pus—a probe passed down proved the bone to be denuded for some extent. He was bled to the amount of 16 ounces, which relieved him. He became more tranquil though still delirious. Towards the evening all his symptoms became aggravated, his pulse rose to 116, hard and full; the skin remarkably hot, and the bowels constipated; he was again bled to the amount of 12 ounces, and a dose of senna mixture given to him. 15th. He was not so restless this morning, but lay perfectly unconscious of what passed around him. No discharge from the wound, but from the ear a large quantity of pus was flowing—bowels freely opened. About 3 o'clock in the afternoon he had a convulsive fit, which continued a quarter of an hour, followed by profuse perspiration; at 7 in the evening he had another fit, after which he sank rapidly, and died at half-past nine. As we frequently find, after death the true history of his case was *now* made out. His mother stated that he had been the subject of discharge from his ear whilst an infant—that for the last few years it had been constant, and of a most offensive smell. During the same time that this discharge existed, he had suffered most severe pain in his head, at times so great as to make him frantic. These symptoms had all been much worse within the last twelve months, with occasional bleedings from his ear; for the last fortnight she said he had scarcely ever been free from pain, and at intervals was quite furious: they had also oberved within the last-named period the external swelling.

*Sectio cadaveris.* The periosteum was found to be separated, from a considerable surface of the squamous portion of the temporal bone, and in the immediate neighbourhood the temporal muscle was sloughy—in the interior an abscess existed between the dura mater and the same part of this bone extending likewise upon the petrous portion — the cavity was circumscribed and contained about half an ounce of very fetid pus. The left cerebral hemisphere, immediately corresponding with this disease, contained an abscess as large as a hen's egg—soft—undefined — containing fetid pus, and broken down brain. The structure of the brain was disorganized to a considerable distance around in the immediate vicinity of the internal abscess — the process had proceeded to the greatest extent—the membranes and cortical part having become green, and so soft as scarcely to bear handling. During the examination the posterior extremity of the right hemisphere was cut off; from the ventricle thus opened a quantity of pus escaped: the usual horizontal section was therefore made, when, both lateral ventricles were found full of matter, the source of which was most satisfactorily traced to the abscess of the brain above described. There was no distinct aperture of communication, but the disorganization had gone on, till the inferior corner of the right ventricle was laid open to the abscess—the foramen commune anterius was distinctly shewn to be the passage by which the pus got into the left ventricle—several long fungous granulations filled up the meatus auditorius externus, and protruded externally —the lower part of this canal was therefore cut away, as far as the membrana tympani, when the granulations were found to be growing from that membrane; their attachment was so intimated, that there can be no doubt they had existed a great length of time. Some pus was found in the cavity of the tympanum, but no communication could be detected between the abscess and this cavity—the viscera of the other cavities healthy.

CASE 3. *Apoplexy from the Rupture of an Aneurism of the middle Cerebral Artery.*

Elizabeth Hancock, æt. 65, a woman of en-bon-point, was brought to St. Thomas's Hospital, August 8, 1830. When admitted she was unable to speak, had hemiplegia of the right side, and was very lethargic—the account given respecting her was, that ten days previously, whilst hanging out clothes, she fell down suddenly in a fit of apoplexy, without any premonitory symptoms of the attack. During the ten days before admission she had been gradually improving, but there were still symptoms of pressure on the brain. She was quite sensible to what was addressed to her, though unable to reply—the hemiplegia was complete, and she had considerable loss of sensation—she was cupped on the back of her neck and head to 16 ounces—a blister applied to the neck, and ordered to take pulv. scam. c. hydr. gr. xv. statim, et hydr. submur. gr. v., 6tis hor. On the 19th she was somewat improved, but complained of pain in her head, referred to the left side. She explained this by putting up her hand — eighteen leeches were applied to this part—and cold lotion constantly.

10th. She complained of increase of pain—15 leeches were therefore ordered, and her bowels having become greatly purged, she was to take infus. catechu ℥ii. urgente diarrhœa.

11th. Sixteen more leeches were applied—the head shaved, and a blister to the vertex—during this time she had been improving gradually—and had so far recovered as to utter a few words. On the 13th she was so much better that she was allowed to sit up in a chair, and to have on her clothes. After being in bed a couple of hours, the sister's attention was drawn to her in consequence of her stertorous breathing, and fixed, open eyes—in fact she was in a fit: it did not, however, continue longer than ten minutes—twenty leeches were applied, and aperient medicines given to her, but at six o'clock in the morning of the 14th another apoplectic attack supervened, during which she died.

*Post mortem examination, seven hours after death.* The surface was much blanched. The calvarium and dura mater being removed, some fluid deeply tinged with blood escaped. A large surface of the left hemisphere was covered with blood effused beneath the arachnoid, and following the course of the middle cerebral artery — a small quantity was also effused external to the membrane — the whole of this hemisphere felt boggy, as if full of fluid—the brain was removed, and the ventricle laid open from the base. This cavity was then found filled with blood, partly fluid, partly coagulated, the whole cerebral surface in contact with which had a soft and ragged texture—the quantity of blood was about half a pound. The branches of the circle of Willis were then removed, in doing which a firm coagulum, the size of a hazle nut, which had been imbedded in the cerebral substance was drawn away—tracing these branches carefully a small aneurism was discovered connected with the middle cerebral artery of the left side, and precisely at the point of its division into branches. To this the coagulum just mentioned was firmly attached — somewhat nearer to the trunk of the artery, but still *in* the aneurism, a rent of about a line and a half in length had taken place, through which doubtless the last and fatal hæmorrhage had occurred—there was not a speck of bone or other diseased appearance in any branch of the arterial circle—no other part was examined.

DELTA.

### LXVIII.

### ST. THOMAS'S HOSPITAL.

### INJURIES OF THE HEAD.

CASE 1. *Slight Concussion of the Brain.—Contused Wounds of Scalp, the Periosteum not denuded.—Erysipelas of Head and Face.*

Samuel Ward, ætatis 44, a horse-keeper in the employ of Messrs. Barclay, the brewers, was struck down in the affray at the Borough election on the 23d November, and was brought into hospital immediately. The men who conveyed him here stated that he had received a heavy blow on the occiput with a thick cudgel, whilst his head was uncovered, from which he fell instantly; and when he lay upon the ground he was kicked and by other means hurt about the head. There was a tolerably clean wound, two inches in length, to the right of the occipital protuberance, in direction from before backwards; there was some contusion around the edges of the wound, and a great deal of blood had flowed from it; the pericranium was uninjured. Two smaller and more ragged wounds were situated on the left side and on the vertex, and from these free hæmorrhage had occurred; the parts around them were also swollen. The occipito-frontalis tendon was not divided in either. No depressing effect was now left on the sensorium, the senses appearing all perfect; the intellect was not confused, and the surface of the body was warm and natural, the first stage of concussion having passed off. He spoke of no pain in his head but that which proceeded from the wounds—there was no vomiting or sickness—pulse accelerated to 90, but natural in its beat and regular. The patient coughed, as if from chilliness; he said he had not been subject to this symptom. A cold bread poultice was applied to the shaven scalp by the dresser's direction, hæmorrhage having speedily ceased spontaneously.

*Vesperè.* Patient has been perfectly quiet, and inclined to sleep—has had some pain of head, and his skin is rather hot—answered to questions quite coherently, but appears listless and dull —his eyes have lost the natural degree of expression, but the pupils contract and dilate naturally—pulse 100, soft, and regular—slight nausea during the day, but no vomiting. Some house-medicine has been given, which has not yet operated, and his head is properly elevated on pillows.

Nov. 24th. Got but little sleep in the night, and has had some pain in his head constantly, though not severe—lies on his belly, and appears much more dull; indeed, he may be said to be in a state of slight stupor—pulse 100, not hard, or full, or irregular—skin moist, rather hot—bowels opened from the physic—wounds are healthy in appearance, and yield a purulent discharge. Mr. Tyrrell ordered ℥xvj. of blood to be abstracted from the nape of the neck by cupping.

About twelve o'clock at night, the pain of head having increased, the skin become dry and more hot, and the pulse more full, with restlessness and great confusion of ideas. Twelve ounces of blood were drawn from the arm, and it afforded very great relief.

25th. Had some sleep; pain of head continues, and also that in the wounds, which have unclean edges, disposed to sloughing, and there is tenderness around them on pressure. Patient is now free from incoherence, but lies drowsy and seems averse to answer when spoken to—pulse 90, natural in its beat—tongue clean, and moist—bowels open. A hot linseed poultice was now applied to the wounds four times a day.

26th. Passed a very restless night, and is considerably worse this morning —he is quite sensible, but drowsy, yet unable to sleep—countenance pale and distressed—pulse 120, very small and feeble—tongue still clean and moist, but there is dryness of the fauces and thirst—sickness but not vomiting—skin cool and dry—wounds of scalp very painful, their edges are sloughing but not to any extent—the pain of head is less, but has continued during the night. Mr. Tyrrell prescribed cal. gr. j., ant. tart. gr $\frac{1}{4}$, 6tis horis.

27th. Rested a little last night, but has suffered a great deal of pain in his head—pulse 120, not so feeble as yesterday—tongue clean, but dry; fauces and mouth dry—thirst increased—skin cool. He is less inclined to sleep, and his appearance is, upon the whole, improved—bowels open.

28th. Pain of head and scalp continues equally severe, but the man is much better—he slept a little in the night, and was refreshed by it—has no drowsiness or stupor—pulse 100, of more power, and regular—less dryness of fauces and tongue.

29th. Erysipelas has appeared around the wounds, and occupies a considerable portion of the face, the affected parts of which are much swollen; it extends forwards to the eyes and posterior border of the cheeks. The wounds afford no discharge, but their edges are not more sloughy. Patient was delirious all the night, and is now so, but not violent—pulse 118, irregular, the beats at one time feeling dilated under the finger, and contracted at another, and compressible by the slightest force; tongue generally white, but not very dry; bowels freely open. Mr. Tyrrell ordered eight leeches to each temple. The calomel and ant. tart. to be taken every four hours.

9, p.m. Face more swollen and involved to a greater extent in erysipelas —delirium unabated, so that he is confined by a straight-waistcoat—pulse unaltered since morning—tongue become rather yellow, and dry—mouth also more dry. Around the wounds on the scalp a soft doughy feel is communicated to the fingers on pressure, as if matter or sloughs were formed beneath. This sensation is not, however, very perceptible; but Mr. Green, who was requested to visit the patient at this time, in consequence of the impression conveyed to his fingers, and from his experience of their good effect in these cases, recommended two incisions to be made about two inches long, near to the wounds, and down to the pericranium. This was done, and a small quantity of pus escaped from the incisions. Rather more than twelve ounces of blood were also thus obtained, as the cuts bled freely.

30th. Still equally delirious, and the erysipelas has spread on the face—pulse 120, rather more feeble. He was for a short time quieter after the incisions of last night, but has been continually talking aloud and incoherently—the extremities have been cold for the last few hours—bowels not open since yesterday morning. Mr. Tyrrell ordered him to have immediately, ammon. subcarb. gr. x., mist. camph. ℥iss.—half a pint of mist. sennæ co. to be thrown up the rectum, tepid—with the following draught every two hours : ℞. Ammon. subcarb. gr. vj., Tinct. hyoscyami, ♏ v., Mist. camphoræ, ℥j. The calomel and tartarized antimony have been this morning omitted.

9, p. m. A sudden loss of power has come on within the last two hours—there is less redness of the parts inflamed, and a death-like pallor over the rest of the countenance—difficulty of breathing has come on, and this is loud, and the patient lies with his mouth some way open—he is singing at intervals loudly, and the delirium is undiminished—pulse very low. An abundant stool has been brought away by the injection. Remedies continued.

Dec. 1st. He continued in this state for nearly two hours, the pulse becoming weaker and the extremities colder, when some porter and about an ounce and a half of brandy were given to him, after taking which his pulse rose and he fell asleep instantly, and had an hour's rest; he shortly slept again for three hours, awaking about 8 o'clock this morning composed, and nearly free from delirium. He now lies quiet, and is capable of answering consistently; says he has some pain in head—surface of the trunk and extremities warm—countenance tranquil, and respiration much easier, though loud—pulse has more power than since delirium set in, 120—tongue dry over all its surface, but not furred, or discoloured—gums are a little turgid from the calomel—bowels open. Mr. Tyrrell gave orders that he should have ℔iss. of porter in the day, to be given at intervals, and not more than half a pint at one time; this being the accustomed stimulus of the man when in health, and used to great excess, was preferred for that reason. Perg. c. haust. 2dis horis.

10, p.m. The powers of the system have a little flagged during the last few hours, and the pulse is 126, weaker, and tremulous under the finger—he has kept up during the day well—breathing rather more laborious, and louder—surface warm—lips and fauces drier, and sordes are beginning to collect about the former and teeth—has just had a little brandy, which has revived him in some degree; has consumed nearly a pint of porter, but taken no other nourishment. The bowels have been opened two or three times in the day, but they were not purging stools. The medicine has been taken regularly.

2d, 1 o'clock, a. m. Has been evidently sinking, but his pulse and strength have been in some measure kept up to this time by brandy given in gruel at repeated intervals.

This stimulant was continued during the night, but coma soon supervened, and he was then unable to swallow any liquid. The breathing became weak and irregular, and the pulse so indistinct that for some time before death, which took place about 9 o'clock this morning, it could not be felt at the wrist.

*Inspection of the body 28 hours after death.* The scalp surrounding the wounds was greatly thickened, and a small quantity of pus existed between their edges, which were sloughy. Upon removing the calvarium, the sinuses of the brain were found in a most turgid state, the dura mater itself being rather vascular, (that if, its venous branches were distended,) and adhering firmly to the bones. About two ounces of serous effusion were observed between the arachnoid membrane and pia mater.

The arachnoid was free from opacity or deposit. The vascularity of the pia mater was increased, or, more strictly speaking, its veins were more turgid than natural. Between the anterior and middle lobes of the cerebrum some extravasation of blood had occurred, from the injury. Little or no effusion had taken place in the ventricles.

There were slight adhesions of the lungs to the pleura lining the chest, and some hard tubercles existed in the upper lobe of the right lung, but they could not be considered sufficient to interfere with the functions of these organs.

Mr. Tyrrell observed on this case, in a clinical lecture, that it might be regarded as one of unusual interest. At first it was one of slight concussion of the brain, complicated with external injury; then erysipelas supervened, accompanied by delirium somewhat peculiar in character. This delirious state, in his opinion, very probably arose from a deficient supply of blood to the brain, the consequence of a want of the accustomed quantity of stimulus to the system. To distinguish delirium originating in inflammation of the membranes of the brain or its substance, from that produced by inequality or irregularity of circulation through the cerebral organ, requiries doubtless considerable tact in most instances, and in many is impracticable. In the latter kind, however, we generally find a pulse, very frequent, but excessively irregular, a few beats being dilated, others contracted, and the current of blood is most easily arrested in the vessel; the patient is not violent; the extremities have a tendency to become cold; and loss of power in the constitution becomes manifest early. And it is also found almost invariably to occur in persons addicted to an extravagant use of ardent spirits, or fermented liquors. With respect to the action of remedies upon this kind of delirium, it is likewise ascertained that the exhibition of stimulants, and especially those of which the patient has been in the habit of making use, is attended with the most unequivocal success. In the present case, we find on the 29th November, the day erysipelas and delirium set in, it is reported that the patient is not violent, and the character of the pulse then specified was very marked. From this condition of the pulse and the kind of delirium, Mr. T. thought that the case would do well under the employment of stimulants at a later period. Illustrative of this treatment, he related three most interesting cases which had come under his care at this hospital, and on which he founded his opinion. The first was that wherein amputation of a limb had been performed 48 hours after a severe injury. The stump became sloughy, and in 62 hours from the loss of the limb delirium was established, unaccompanied by violence; the pulsations of the artery at the wrist were now dilated, now contracted, and the flow of blood in the vessel was readily obstructed. The man having been largely addicted to spirits, porter and gin were ordered. Sleep was immediately obtained; delirium subsided in a surprisingly short time; the pulse became regular, and of more power; and the case from that period went on well. In the second, which was an example of diffuse inflammation of the cellular tissue, mortification was threatened, and indeed vesicles had formed on the limb. The patient had delirium soon after this, of the same character, and a pulse also of s similar kind. By giving gin and brandy, which were the common stimuli of his patient, these symptoms disappeared. The third case mentioned was a compound fracture of the lower part of the humerus; the bone was much comminuted, and the fracture extended into the elbow-joint. The surface of the body was cool, and the patient had most remarkable self-possession; when amputation was alluded to, he seemed quite unconcerned, and left every thing to the decision of Mr. Tyrrell, expressing his fullest consent to whatever Mr. T. might judge proper. Seeing the great command the man had over his feelings, Mr. T. thought this decidedly in his favour, and determined on making an attempt to preserve the limb. Proper means were carefully taken, as the importance of the case demanded. The patient was plainly a little intoxicated at the time; but the case went on very well for the first few days, when delirium commenced, and sloughing took place in the wound. This continuing till the next day, Mr. Tyrrell came down to the hospital with his instruments, having resolved on amputating when he heard what state the man was in that morning. The patient's wife being present in the ward when he entered to visit him in almost a hopeless state, as the man was rather tipsy on admission, Mr. T. asked her what habits of life her husband had indulged in, and was told that he had lived very *regularly*. This regularity, however, proved to consist in his taking eleven or twelve glasses of gin every morning before breakfast. On being loudly asked whether he would like to have some gin, the word in his delirious state caught his ear, and it was no doubt recognized as his particular favourite, as he kept on repeating it for several times and spoke of nothing else. Some was therefore instantly put to his mouth, and soon swallowed, together with some porter. It was scarcely taken, when he fell asleep, remained tranquil, the delirium ceased in a few hours, and he ultimately got well; the limb being saved and but slight anchylosis existing.

From these cases Mr. Tyrrell was led to think that the one which formed the subject of the present lecture would terminate in the same way under the use of stimulants. His impression now is that the measures, consistent with the view he had taken of the case, were not adopted early enough. For some time before the patient's death, he prognosticated to the pupils and dressers then by the bed side, that serous effusion would be found generally existing between the arachnoid membrane and pia mater, which we see corroborated by post mortem examination. His reason for expecting this appearance was that the patient had been too long in a state of tranquil delirium, without evidence of active vascular action, for meningeal inflammation to continue; and effusion of serum is known to be the result of inequality of circulation in the brain, which is best relieved by nourishment, and where, as in the present case, the system has become habituated to the common stimulant beverages, by giving these and with freedom. In treating the case, it might be said there existed two different states of the brain; the one, from concussion, and the other from erysipelas. The time for administering stimulants was therefore less obvious than if the erysipelas had been uncomplicated; for where concussion has occurred, we should be very careful that we err not in having recourse to these remedies too early.

Case 2. *Concussion of the Brain.*

James Baker, æt. 20. admitted Nov. 23d, in a state of intoxication, and with his face besmeared with blood, from a blow on the nose, and lower part of face. He had also received a severe blow from a cudgel on the back part of his head, which levelled him to the ground. When admitted, he was very violent, evidently from the effects of liquor, would not be put to bed, nor suffer his clothes to be removed till he was compelled by force. To appearance, he was labouring more under the symptoms of inebriety than those of concussion of the brain, yet he was plainly suffering from the latter. He vomited copiously, and the contents of the stomach smelt strongly of rum and gin. The pulse was very small and feeble, indeed could be felt only at times, and appeared about 110, though we cannot state this with certainty, as from the restlessness of the patient and the nature of the pulse itself we had the greatest difficulty in making any enumeration by the watch. Pupils were dilated, and not responsive to light—the surface of the body was warm and moist. Pressure at the lower part of the occipital bone produced pain, indicated by his suddenly rising up from the pillow, as if to enquire after the cause of his suffering; but no injury of bone or soft parts could be detected.

4, p. m. Somewhat recovered from his drunken state, and the pulse is more full and distinct than in the morning, but excessively small, not more than 70. The pupils contracted, upon a candle being brought near to them. He moans occasionally, from pain in the head. The occiput being now shaved, a contusion is observable at its lower part, and exquisite pain seems excited by making pressure upon it—has vomited some more offensive matters. The head is elevated on a pillow, but he is very restless.

10, p. m. He is rather more sensible and quiet, but otherwise there is no alteration since the afternoon—has been somewhat drowsy, and is now dozing—the face is hot and red—ordered, Cal. 5 grains immediately, to be followed in three hours by a dose of house medicine. This was, however, instantly returned by vomiting, and when repeated the following morning was again thrown up as soon as taken.

Nov. 24th. Restless throughout the night, and evidently suffering pain in his head. He now lies in some degree insensible, being aroused with difficulty, but the answers he does give are perfectly rational. He moans a good deal, and the pain of head appears increased—cheeks red but not hot—pulse very small and irregular, just distinguishable, 90—pupils capable of acting—no motion from the bowels. Mr. Tyrrell directed cupping from the back of the neck to ℥xvj. and if necessary in the evening, blood then to be drawn from the arm. His reasons for employing blood-letting were derived rather from the general symptoms of injury to the brain, such as confusion of his intellect, the continuance of pain in head, &c. than from the state of the pulse. ℞. Cal., gr. v., Ext. Col. co., gr. x., statim.

25th. Pain of head increasing, and the skin beginning to get hot, ℥x of blood were taken last night about eight o'clock. He seemed in some degree more sensible after the bleeding, and passed a few hours in a dozing state, not to be called sleeping, during the night. Lies this morning in nearly the same state as to drowsiness, but answers more readily—has had no vomiting—complains only of pain in head, which appears unrelieved—pulse small, 70, and irregular — skin dry, but not hot—tongue white, and furred—bowels opened for the first time at six o'clock this morning, a dose of physic being given last night as the colocynth and calomel had not then acted.

26th. More sensible, and more easily aroused—is constantly dozing, but mutters not, nor moans, and appears free from pain—he, however, complains of pain continuing in his head, but says it is less—pupils are at present very much dilated, nearly to the whole extent of the surface of the iris—pulse more easily felt, 62 and regular—tongue white and dry—skin temperate—bowels twice opened since last report, the dose of medicine being repeated last evening. The lower part of his face, which was a little swollen, has subsided, and no ecchymosis is left. He still flinches on pressure of the occiput, but less than he did—no contusion is now visible.

27th. Sensibility increased — less drowsiness — pupils not so dilated as yesterday — pain of head much diminished — pulse 62, has more power — tongue cleaning.

28th. Improvement in every respect since yesterday.

29th. No pain of head — pulse very small, and felt even with more difficulty than a few days ago, 62—has got rid of all somnolency—bowels open.

Dec. 1. No symptom of injury or mischief within the head is now observable, but the pulse continues in the same very low and small state, and is rather under 60.

3d. Pulse has become more full and distinct, and is perfectly regular and compressible at 64—slight increase of pain of head has come on this morning — tongue white, but moist — bowels open.

4th. Continuance of pain, which he describes as dull and aching, and felt through the whole head — pulse 70, quicker, and feels no longer oppressed.

6th. Has been during the last two days free from pain of head, and he in consequence very imprudently sat up yesterday for a short time without leave.

9th. The patient has had permission to sit up to day—there is no pain, or giddiness about the head.

14th. Slight superficial swelling, with tenderness on pressure, has taken place on the scalp, but the surface it occupies is small and circumscribed. He has, however, no feverishness. Ordered to have the part shaved and blistered. Confinement to bed, and low diet.

20th. The swelling has nearly disappeared, and the man may be pronounced without ailment.

Upon this case, Mr. Tyrrel observed, in his lecture on the 8th, that although the patient then appeared quite recovered, he should retain him in the hospital some days before he should present him. And the trifling relapse which has lately occurred might certainly have been of serious consequence had the man been at liberty and returned to his employment.

CASE 3. *Contused Wounds of Scalp, with exposure of Os Frontis.*

Ann Hayman, æt. 39, admitted into Queen's ward on Thursday, the 2d Dec. She has two wounds of the scalp, near the left temple, between this and the median line. One (the larger) is at the junction of the hairy scalp with the forehead; and the other is on a line parallel with this, and situated about an inch and half higher up on the scalp. The former is horizontal and about an inch in length, and intersected in the centre by an obtuse vertical cut; the latter is not more than half this size. Their edges are irregular, and in a state not at all favourable for adhesion; those of the wound on the forehead, indeed, are slightly sloughy, and severed to their utmost extent. There is a tolerably free discharge of healthy matter from them, and the bone is denuded at the bottom of both. From the smaller one of the two some very minute pieces of mortar, which the patient considered bone, have come away with the discharge. These wounds were received, we are told, on Saturday last, when the patient fell down twelve or thirteen stairs, and her head came in contact with an angular-shaped post at their foot, by which their irregular figure was occasioned. She was insensible for an hour afterwards, but did nothing more that night than foment her head with hot water. There was also free hæmorrhage from both wounds at the time. The next day (Sunday) she had considerable pain of the head, but no want of sensibility, or confusion of her intellect. On Monday she applied at the surgery of this hospital, and was seen by Mr. Tyrrell with the rest of out-patients. He enjoined perfect quietude, and ordered some purgative medicine and calomel at night, and poultices to the head; but did not think her present state called for bleeding. Since then, the pain of head as continued and rather increased than otherwise; she has had vertigo occasionally, with confusion of ideas, and her memory has begun to fail; the sense of hearing is perfect, her sight clear, and pupils of natural dimensions. During the last few days she has had rigors, accompanied with chattering of the teeth, at frequent intervals; and she has in the mean time felt a constant chilliness, so that the surface could not be kept warm by clothing. No heat of skin, or sweating, followed the rigors. The pulse is 88, rather full, but not hard or irregular; skin in some degree above natural temperature; tongue whitish, not drier than natural; restlessness, and she has had no sleep for several nights; bowels rather confined. Mr. Green ordered as follows on the 3d, when the patient was in the above condition. V.S. ad ℥xij. Cal., gr. j. o. n. Magn. Sulph. ʒj., Vin. Ant. Tart. ♏xx., Mist. Menth. ℥iss. 6tâ q. horâ. Fever Diet. Head to be shaved around the wounds, and to these, cat. lini.

Dec. 4. The bleeding produced a little disposition to faintness. She thinks the pain of head is lessened by it, but not to the extent that can say she is relieved, for she slept not better last night than any preceding—pulse 80, reduced in fulness, and quite soft and compressible—has had less chilliness—bowels open twice.

6th. The expression of the patient's countenance is rather altered, and there is a slight irregularity in the motions of the eye-ball, the pupils being dilated to some extent. She has not obtained more than a few hours sleep, interrupted at short intervals, during the last two nights. But in the day-time of yesterday she had a continued nap of some hours. The surface has felt chilly, and last night a rigor, equal in severity to any of the former, took place; it was preceded by increase of pain, and attended by a sensation as if cold water were poured upon the head and back. It lasted above an hour, and at its termination the head was rather easier. No increased heat, or sweating, succeeded it. The pain at other times has been nearly the same in degree, and constant—pulse 84, rather full, but not hard—skin feels to the hand temperate—tongue cleaner considerably, and moist—bowels open. Wounds are in no pain, and discharge copiously—a few pieces of hardened mortar have latterly found their way from the bottom of the wounds, very similar in appearance to dead bone.

7th. Passed a somewhat easier night than the preceding, but had very little sleep—has experienced neither rigors, nor chilliness—pain principally in the front of the head, and some throbbing and giddiness—does not seem to express herself quite clearly—pulse 80, soft—bowels opened two or three times a day—wounds look well, and disposed to granulate.

8th. In consequence of prurigo of the whole body, which she says she has experienced for some days, attended with slight efflorescence, the patient went into a warm-bath last night by desire of Mr. R. Whitfield, the assistant apothecary, and this morning she states her head is not worse, but on the contrary, less painful. The surface of the body, she also says, now feels warm for the first time. Her pulse is increased to 88, and is decidedly hard.

Dec. 9th. Reports herself much better in every respect. The pulse has to day returned to 80, and its former softness.

11th. No pain of head, or vertigo—surface feels warm—pulse 76, soft, and regular—calomel omitted—mouth not affected.

14th. Has sat up daily, without any return of former symptoms—wounds almost healed.

19th. Wounds nearly cicatrized, and no unpleasant symptoms about the head remain.

CASE 4. *Compound Fracture of the Cranium, unaccompanied by the usual symptoms.*

We have taken this case from the practice of Guy's Hospital, thinking that it cannot be better placed than in the present report of injuries of the head, from the neighbouring hospital.

William Smith, aged 10 years, whilst crossing the road, was knocked down by a horse, and received from the animal a kick upon the head. He was stunned by the blow, and in that state, which lasted ten minutes, was carried to the Deptford Infirmary, close to which the accident happened. Some light dressing was applied to the seat of injury, and he was thence forwarded in a cart to the hospital, where he arrived about 11 o'clock, on the 2d October, 1830. At this time he was faint, apparently from loss of blood—pulse about 60, and small—hands and feet cold—countenance pale, but not expressive of much suffering. Pupils much dilated, but readily responsive, we cannot say actively so, to the stimulus of light. He had not vomited, and possessed the usual command over his mental powers, answering unconfusedly any questions put to him.

Being placed in bed, the dresser removed the applications from his head. A small, but deeply incised wound presented itself, situated about two inches above the meatus auditorius externus. The blood had then ceased to flow, but the friends informed us that the quantity lost was very considerable. On close examination it was very apparent there was a fracture of the left os parietale, and also that the fractured portion was depressed. There were besides several slight bruises on other parts of the scalp. The little boy was seen by Mr. Key about an hour after his admission; he had then in a great measure recovered from his state of collapse, and was ordered into the operating theatre. The external wound was first enlarged by the scalpel, and this dividing some branches of the temporo-occipital artery, three ligatures were required to restrain the bleeding. A depressed piece of bone was found, of circular shape, and nearly the size of a shilling;

Mr. Key tried to draw this out with a pair of dressing forceps, as it was perfectly free and moveable from the surface, from which it had been detached; but the inner table being fractured to a much greater extent than the outer, he could not succeed in this attempt, as the external apperture of the cranium was not large enough to admit the extrication of the obliquely fractured portion of the inner table, which was driven in for some distance, and consequently pressing against the brain by its whole surface. Having ascertained the cause of the difficulty, Mr. K. sent for a strong pair of bone forceps, and by dividing the bone with these, the two portions were extracted singly, and the operation was then completed. The pieces of bone, which were shewn a few days afterwards to Sir A. Cooper, at first sight explained the nature of the fracture. No wound of the dura mater could be seen, but the patient lost much blood during the operation, from the laceration of a branch of the middle meningeal artery. The bleeding, however, ceased, when the boy ceased to cry. A small piece of lint was applied to the wound, and over it a linen compress, to be kept well wetted with lotio plumbi.

The boy was tolerably comfortable throughout the day, and passed a good night, continuing in this condition till about one o'clock the following day, when he became somewhat uneasy. His pulse, which since the operation had ranged between 80 and 90, advanced beyond 100, and had increased both in frequency and power at three o'clock, when we found him with a remarkably hot and dry skin, the pulse 126, and hard—there was slight strabismus, the left eye being turned unnaturally inwards — he was exceedingly restless, and complaining of great pain in the forehead. His bowels had been twice freely moved by calomel taken in the morning. Looking upon these as symptomatic of inflammation, or at least as premonitory, the dresser took blood from his arm to the amount of six ozs. and this flowing freely, a short state of syncope was obtained. Three hours after the bleeding 15 leeches were applied to the forehead, as the pain, though mitigated by the venesection, was again returning; and a purge of calomel and jalap was given. The pulse was at this time reduced to 85, but intermitting. Bread and water poultice to the wound.

Oct. 4th. Was relieved greatly by the leeches. Has had much good sleep in the night, and is quite easy this morning, yesterday's train of unfavourable symptoms having nearly disappeared. Two evacuations have been procured from the bowels. A small quantity of pus is observed in the wound, which has a healthy appearance.

The little patient went on well daily. In a few days the wound presented very healthy granulations; although at the bottom of it a considerable portion of denuded bone was to be seen for some time. No exfoliation occurred, and granulations sprang up from the lowest part. These cicatrized but slowly, and on the 15th November a very small and healthy wound remaining, the boy left the hospital, having been within its walls five weeks.

